# Syphilitic uveitis misdiagnosed as viral retinitis—a misleading history

**DOI:** 10.1186/s12348-018-0164-5

**Published:** 2018-12-04

**Authors:** Manisha Agarwal, Richa Ranjan, Lagan Paul, Deepa Sharma

**Affiliations:** 1 Dr. Shroff’s Charity Eye Hospital, 5027-Kedar Nath Road, Daryaganj, New Delhi, 110002 India; 2 Dr Ram Manohar Lohia Hospital and Post Graduate Institute of Medical Education and Research, Baba Kharak Singh Marg, New Delhi, 110001 India

**Keywords:** Syphilis, Uveitis, Human immunodeficiency virus, Retinochoroiditis

## Abstract

**Background:**

Syphilitic uveitis is an infective uveitis and a great mimicker. Misdiagnosis can lead to delay in the specific treatment resulting in deterioration of uveitis and loss of vision.

**Findings:**

A 38-year-old unmarried female presented with pain, redness, and blurring of vision in the left eye for the last 5 days. She denied history of any sexual exposure in the past. Anterior segment examination of the right eye was normal and the left eye showed keratic precipitates with anterior chamber cells and iris pigments on anterior lens capsule. Fundus examination of the right eye showed a hyperemic disc with posterior placoid retinochoroiditis and the left eye showed dense vitritis, hyperemic disc, and superficial retinal precipitates. She was misdiagnosed as viral retinitis elsewhere and started on antivirals with oral corticosteroids which resulted in deterioration of uveitis and progression to bilateral involvement. Further systemic investigations confirmed the diagnosis of syphilis and human immunodeficiency virus infection. She was then started on anti-syphilitic and anti-retroviral therapy which resulted in restoration of the vision in one eye.

**Conclusion:**

Syphilitic uveitis does not occur in primary disease and is common in secondary and early latent phase of syphilis. History given by the patient is often contributory however at times can be misleading. A high clinical suspicion and thorough investigation is necessary for the correct diagnosis and timely intervention in preventing loss of vision.

## Introduction

Acquired syphilis is a sexually transmitted disease caused by spirochetal bacterium *Treponema pallidum*. It affects most of the organ systems including skin, heart, blood vessels, bone, nervous system, and eye [1, 2]. Ocular syphilis has varied manifestations and may present as anterior uveitis, posterior uveitis, panuveitis, retinitis, papillitis, and even scleritis, making it a “great masquerade” [3]. It may result in visual loss if diagnosed and treated as a non-infectious uveitis. We report a case of syphilitic uveitis misdiagnosed as viral retinitis resulting in loss of vision in one eye.

## Case report

**Fig. 1:**
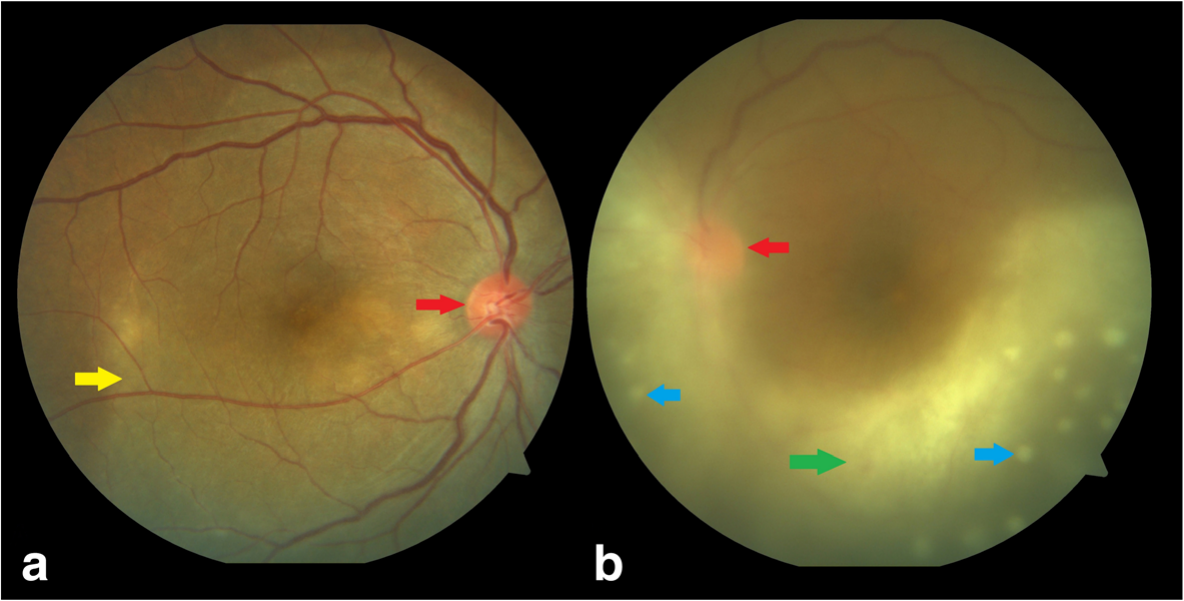
Color fundus photo of **a** the right eye showing a hyperemic disc (red arrow) with posterior placoid retinochoroiditis (yellow arrow) and **b** the left eye showing dense vitritis (green arrow) with hyperemic disc (red arrow) and superficial retinal precipitates (blue arrow)

A 38-year-old unmarried female patient presented with pain, redness, and blurring of vision in the left eye for the last 5 days. She had a history of hypothyroidism, recurrent ulceration of breasts, hair loss, nausea, vomiting, and gastric pain. She was on treatment for hypothyroidism, anemia, and esophageal reflux disease. She denied history of any sexual exposure in the past. On examination, the best corrected visual acuity (BCVA) in the right eye was 6/6, N6 and in the left eye was hand movement (HM),< N36. Intraocular pressure (IOP) was 14 mmHg in the right eye and 16 mmHg in the left eye. Anterior segment examination of the right eye was normal and the left eye showed keratic precipitates, anterior chamber cells 2+ with flare (SUN—standardization of uveitis nomenclature grading) [4] and iris pigments on the anterior lens capsule. Fundus examination of the right eye showed a hyperemic disc with posterior placoid retinochoroiditis and the left eye showed dense vitritis with hyperemic disc and punctate yellowish lesions suggestive of superficial retinal precipitates (Fig. 1).

She was advised investigations but was lost to follow-up for 1 month and diagnosed elsewhere as viral retinitis. Investigations done showed decreased white blood cell (WBC) count—3980 mm^3^ and raised erythrocyte sedimentation rate (ESR)—35 mm/h. Mantoux test − 0 mm induration after 72 h. She was started on oral valacyclovir 1 g three times a day with topical prednisolone acetate 1% and oral corticosteroids 1 mg/kg weight which she was using for the last 2 weeks.

**Fig. 2:**
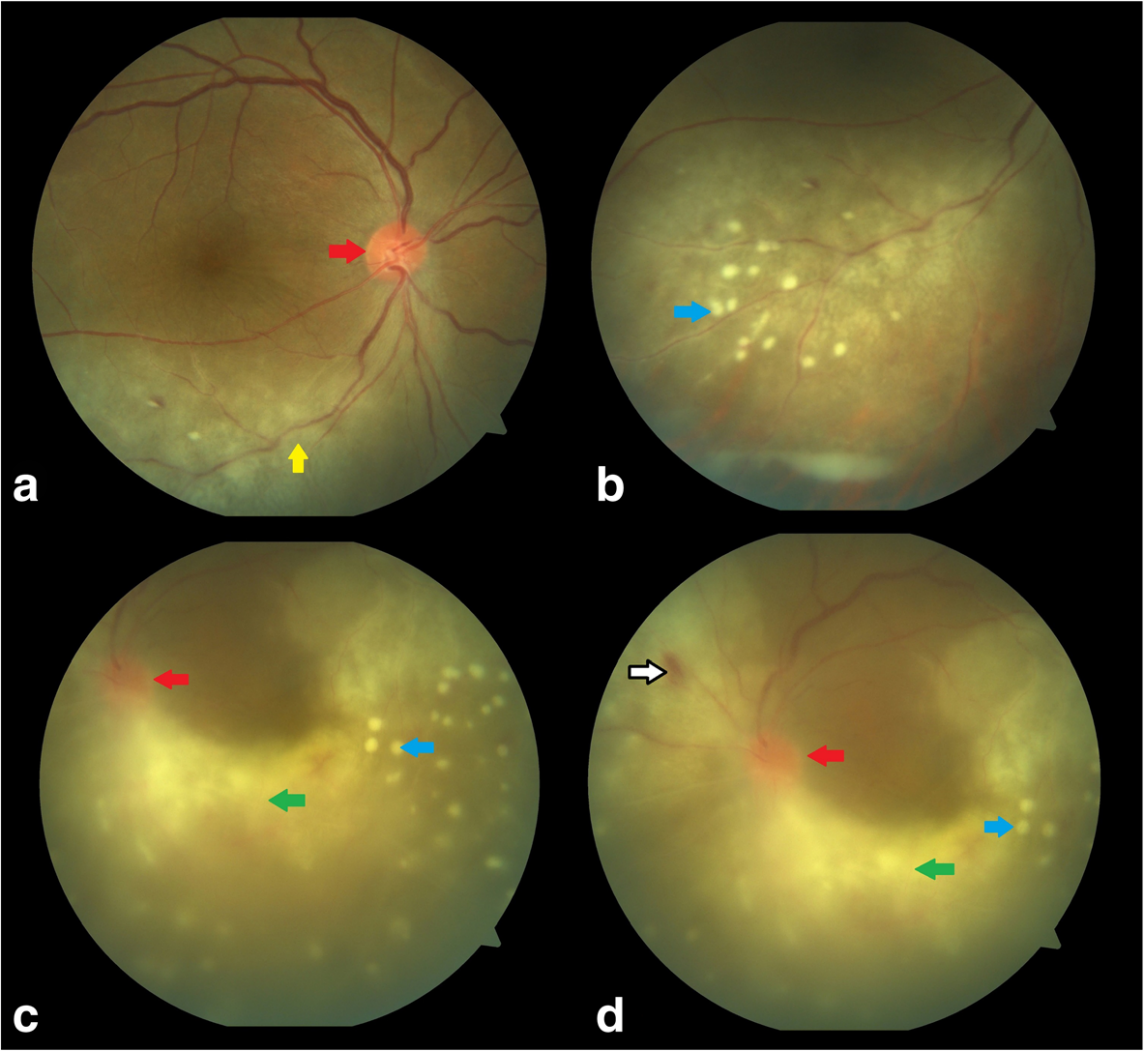
Color fundus photo of **a**, **b** the right eye showing hyperemic disc (red arrow) with ground glass retinochoroiditis (yellow arrow) and superficial retinal precipitates (blue arrow) along the inferotemporal arcade and **c**, **d** the left eye showing dense vitritis (green arrow) with hyperemic disc (red arrow), intraretinal hemorrhage (white arrow with black border) superonasal to the disc, and superficial retinal precipitates (blue arrow)

**Fig. 3:**
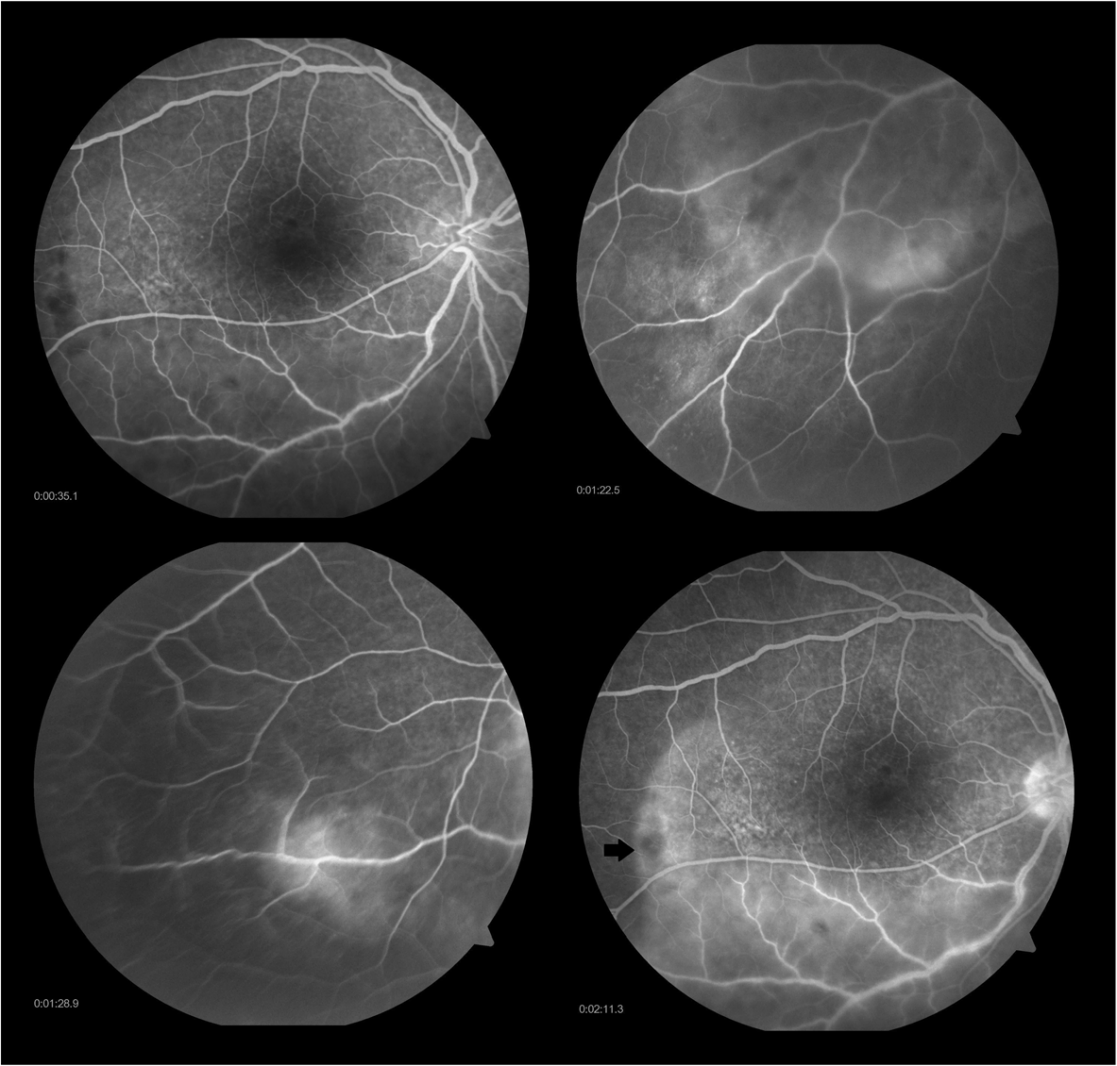
Fundus fluorescein angiography of the right eye showing a hot disc with a hyperfluorescent edge of the posterior placoid retinochoroiditis lesion (black arrow)

**Fig. 4:**
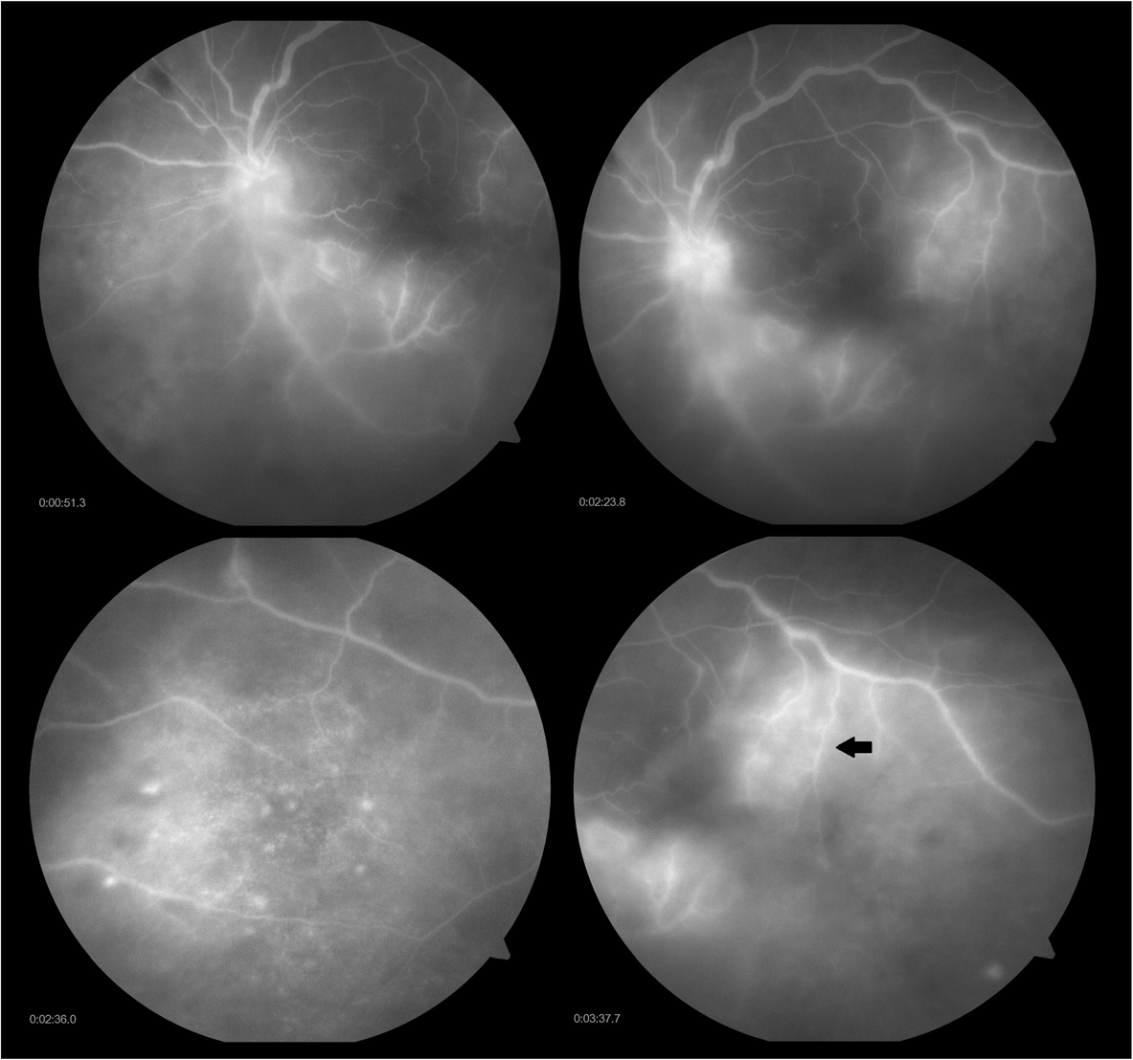
Fundus fluorescein angiography of the left eye showing perivascular leakage suggestive of active vasculitis (black arrow)

**Fig. 5:**
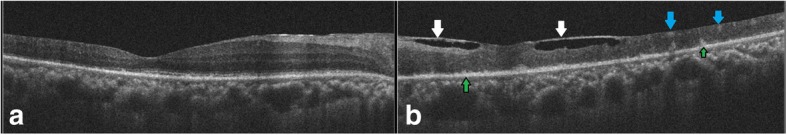
Optical coherence tomography of **a** the right eye showing a normal foveal contour and **b** the left eye showing epiretinal membrane (white arrow) with superficial retinal precipitates (blue arrow) and irregularity of the retinal pigment epithelium (green arrow with black border)

After being lost to follow-up for 1 month, she presented with blurring of vision in the right eye for last 1 week and ocular pain in both the eyes. The BCVA in the right eye was 6/36, N24p and in the left eye HM, < N36. Anterior segment examination showed anterior chamber cells 2+ with flare (SUN grading) in both the eyes, with keratic precipitates and iris pigments on anterior lens capsule in the left eye. Fundus examination of the right eye showed a hyperemic disc with ground glass retinochoroiditis and superficial retinal precipitates along the inferotemporal arcade and the left eye showed dense vitritis with hyperemic disc, intraretinal hemorrhage superonasal to the disc and superficial retinal precipitates (Fig. 2). Fundus fluorescein angiography (FFA) of the right eye showed a hot disc with a hyperfluorescent edge of the posterior placoid retinochoroiditis lesion (Fig. 3) and the left eye showed perivascular leakage suggestive of active vasculitis (Fig. 4). Optical coherence tomography (OCT) of the right eye was within normal and the left eye showed epiretinal membrane (ERM) with superficial retinal precipitates and irregularity of the retinal pigment epithelium (RPE) (Fig. 5). On further investigation, the venereal disease research laboratory (VDRL) test (1:128) and treponema pallidum hemagglutination (TPHA) test were both reactive. CD4 count was reduced—198 cells/microliter. Human immunodeficiency virus (HIV) I was positive and hepatitis A, B, and C were negative. She was referred to the acquired immunodeficiency syndrome (AIDS) cell and started on anti-retroviral therapy (HAART—highly active anti-retroviral therapy) and treated with injection benzathine penicillin 2.4 million international units (IU) intramuscular (IM) weekly for 3 weeks under close observation. Oral valacyclovir was stopped. Seeing to the general health of the patient and underlying HIV infection with intolerance to oral corticosteroids (esophageal reflux disease), the treating physician decided to stop oral corticosteroids. The patient was kept under close observation for the same. Cerebrospinal fluid (CSF) tap was positive for VDRL. Ceftriaxone 1 g intravenous twice daily for 2 weeks was added. Repeat CSF tap analysis after 3 weeks of treatment was negative for VDRL and CD4 count had improved to 319 cells/microliter, following which the anti-treponemal treatment was stopped and the anti-retroviral therapy was continued.

**Fig. 6:**
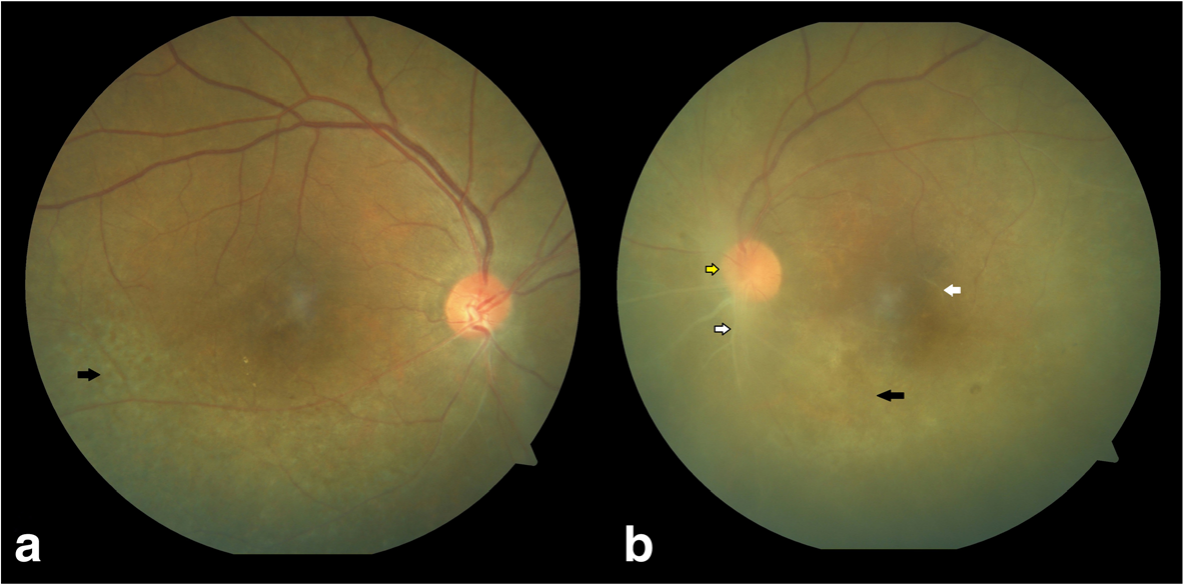
Color fundus photo at 6 weeks follow up of **a** the right eye showing pigmentary alterations (black arrow) along the inferotemporal arcade and **b** the left eye showing disc pallor (yellow arrow with black border), sclerosed vessels (white arrow with black border), pigmentary alterations (black border), and epiretinal membrane (white arrow) at the macula

Follow up at 6 weeks, the BCVA in the right eye was 6/18, N6 and in the left eye finger counting (FC) at 1 m, < N36. Fundus examination of the right eye showed pigmentary alterations along the inferotemporal arcade and the left eye showed disc pallor, sclerosed vessels, pigmentary alterations, and ERM at the macula (Fig. 6).

**Fig. 7:**
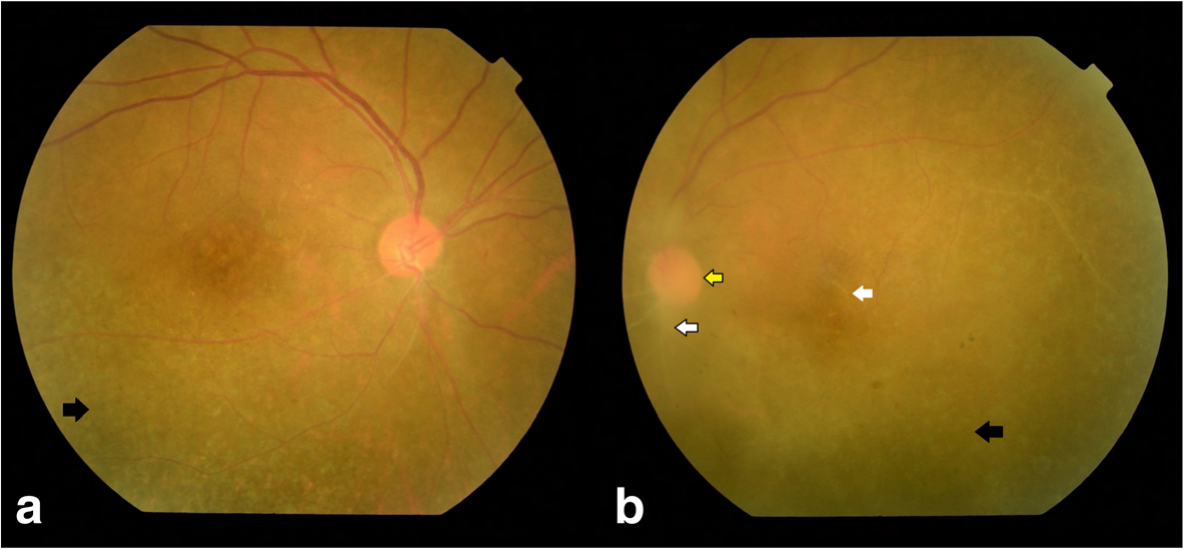
Color fundus photo at 6 months follow up of **a** the right eye showing pigmentary alterations (black arrow) along the inferotemporal arcade and **b** the left eye showing disc pallor (yellow arrow with black border), sclerosed vessels (white arrow with black border), pigmentary alterations (black border), and epiretinal membrane (white arrow) at the macula with no evidence of recurrent active inflammation

Follow up at 6 months, the BCVA in the right eye was 6/12, N6 and in the left eye FC at 3 m, N24. On examination, there was no evidence of recurrent active inflammation (Fig. 7).

## Discussion

Syphilis is a sexually transmitted chronic disease caused by the spirochete *Treponema pallidum*, which may spread via mother-to-child transmission (congenital syphilis) or acquired in adulthood (acquired syphilis). Syphilitic uveitis is an infectious uveitis. It does not occur in primary disease and is the most common in the secondary stage of acquired syphilis.

Ocular involvement by syphilis has varied manifestations such as iridocyclitis, papillitis, retinochoroiditis, retinal pigment epithelitis, serous retinal detachment, cystoid macular edema, vitreous opacity, and neuroretinitis [5, 6]. The most common manifestation being retinochoroiditis, in which infiltration of inflammatory cells from the choroidal capillaries, afflicting the Bruch’s membrane and retinal pigment epithelium, is thought to constitute the pathological progression [7].

Ocular syphilis is a great mimicker [8, 9]. Misdiagnosis may lead to an undue delay in initiating the treatment resulting in deterioration of uveitis and patient’s general health. Our patient was misdiagnosed as viral retinitis and started on anti-viral therapy and corticosteroids elsewhere resulting in worsening with progression to bilateral involvement and a permanent damage to the left eye with no recovery of vision. The clinical signs were very classical of syphilitic uveitis especially the presence of superficial retinal precipitates which are pathognomonic of syphilitic uveitis; however, syphilitic uveitis is more common in the males [10] and our patient was an unmarried female who denied any sexual exposure in the past. This was an important misleading history given by the patient leading to improper investigations and delay in confirmation of the diagnosis. The diagnosis of ocular syphilis leads to the diagnosis of HIV in our patient. As reported in literature, the frequency of syphilitic uveitis in HIV-infected individuals has significantly increased in recent years [11, 12]. Initiation of both anti-syphilitic and anti-retroviral therapy resulted in resolution of the retinochoroiditis and salvaging the vision in at least one eye.

Various risks factors associated with combined syphilis and HIV infection as reported in the literature include homosexual male, age group 18–30 years, multiple sexual partners [13, 14]. Our patient did not fit into any of the common risk factors and also denied history of any sexual exposure which led to delay in the diagnosis.

On review of literature, we found a similar case reported by Zambon F et al. [15], where a case of syphilitic uveitis was misdiagnosed as acute retinal necrosis (ARN) and started on antivirals and oral corticosteroids leading to no light perception in the affected eye.

Diagnosis of syphilitic uveitis or viral retinitis may be difficult in the presence of severe vitritis. Syphilitic retinitis is a form of necrotizing retinitis [6, 16]. The common manifestation is in the form of one or more yellowish white patches of necrosis, often associated with vasculitis, vitreous inflammation, and anterior segment inflammation. This presentation often mimics ARN of herpetic origin. However, in ARN, the necrotic lesions start in the peripheral retina, whereas in syphilitic retinitis they are often located at the posterior pole. In syphilitic retinal necrosis, one has the impression that the surface of the lesion is somewhat indistinct, as if a layer of exudates obscure the underlying retina from view, whereas in ARN one can clearly identify the surface of the lesions as the surface of the thickened, necrotic retina. The retinal necrotic tissue tends to be homogeneous in ARN, whereas the areas of necrosis in syphilitic retinitis have a mottled aspect that becomes even more obvious in the healing phase. Superficial retinal precipitates are pathognomonic of syphilitic retinitis and are absent in ARN. Most often these two entities are diagnosed clinically; however, in case a doubt exists, we may confirm through vitreous sampling and subjection to polymerase chain reaction (PCR).

Syphilitic uveitis shows a good response to penicillin; however, one may encounter an increased ocular inflammation known as Jarrisch Herxheimer reaction after the initiation of the treatment requiring oral corticosteroids. However in our case, the general health of the patient was not good with underlying HIV infection and she was intolerant to oral corticosteroids (esophageal reflux disease); therefore, it was decided to stop them by the treating physician.

We report this case as syphilis which is uncommon in our country; however, there has been a resurgence in the last few years. We routinely investigate our patients based on clinical suspicion and the history given by the patient is often contributory however at times like in our case was misleading, leading to a delay in the management. We need to be aware of the varied manifestations of syphilis and suspect immunosuppression when a patient presents with a zero Mantoux reading in a tuberculosis endemic country like ours. We need to have a high level of clinical suspicion and thoroughly investigate the patient before initiating corticosteroid therapy in an infective uveitis as it may lead to delay in a specific treatment and result in permanent loss of vision.

## References

[1] Margo CE, Hamed LM (1992). Ocular syphilis. Surv Ophthalmol.

[2] Gass JDM, Braunstein RA, Chenoweth RG (1990). Acute syphilitic posterior placoid chorioretinitis. Ophthalmology.

[3] Aldave AJ, King JA (2001). Ocular syphilis. Curr Opin Ophthalmol.

[4] Zierhut M, Deuter C, Murray PI (2007) Classification of uveitis—current guidelines. EUROPEAN OPHTHALMIC REVIEW:77–78. 10.17925/EOR.2007.00.00.77

[5] Kiss S, Damico FM, Young LH (2005). Ocular manifestations and treatment of syphilis. Semin Ophthalmol.

[6] Doris JP, Saha K, Jones NP, Sukthankar A (2006). Ocular syphilis: the new epidemic. Eye.

[7] Kobayashi T, Katsumura C, Shoda H, Takai N, Takeda S, Okamoto T, Maruyama K, Tada R, Tajiri K, Kida T, Ikeda T (2017). A case of syphilitic uveitis in which vitreous surgery was useful for the diagnosis and treatment. Case Rep Ophthalmol.

[8] Puech C, Gennai S, Pavese P, Pelloux I, Maurin M, Romanet JP, Chiquet C (2010). Ocular manifestations of syphilis: recent cases over a 2.5-year period. Graefes Arch Clin Exp Ophthalmol.

[9] Durnian JM, Naylor G, Saeed AM (2004). Ocular syphilis: the return of an old acquaintance. Eye.

[10] Mathew RG, Goh BT, Westcott MC (2014). British ocular syphilis study (BOSS): 2-year national surveillance study of intraocular inflammation secondary to ocular syphilis. Invest Ophthalmol Vis Sci.

[11] Rishi E, Govindarajan MV, Biswas J, Agarwal M, Sudharshan S, Rishi P (2016). Syphilitic uveitis as the presenting feature of HIV. Indian J Ophthalmol.

[12] Wickremasinghe S, Ling C, Stawell R, Yeoh J, Hall A, Zamir E (2009). Syphilitic punctate inner retinitis in immunocompetent gay men. Ophthalmology.

[13] Novak RM, Ghanem A, Hart R, Ward D, Armon C, Buchacz K, Outpatient Study Investigators HIV (2018) Risk factors and incidence of syphilis in human immunodeficiency virus (HIV)-infected persons: the HIV outpatient study, 1999–2015. Clin Infect Dis. 10.1093/cid/ciy348 10.1093/cid/ciy348 PMC11307151 29688270

[14] Chen L, His JH, Wu X, Shen Z, Lu H, Chen H, Huang H, Zhang H, Ruan Y, Shao Y, Tang Z (2017) Disparities in HIV and syphilis prevalence and risk factors between older male clients with and without steady sex partners in southwestern rural China. BMC Infect Dis. 10.1186/s12879-017-2367-z 10.1186/s12879-017-2367-z PMC5389008 28403825

[15] Zambon F, Silva FLN, Sá Cavalcante AF, Nakashima Y, Helal J (2010). Syphilitic retinitis and panuveitis simulating acute retinal necrosis: case report. Arq Bras Oftalmol.

[16] Tran TH, Cassoux N, Bodaghi B (2005). Syphilitic uveitis in patients infected with human immunodeficiency virus. Graefes Arch Clin Exp Ophthalmol.

